# Assessing the applicability of big data driven urban vibrancy analysis in mixed urbanized-depopulated contexts: a case study of a Japanese city

**DOI:** 10.1038/s41598-026-43156-w

**Published:** 2026-03-10

**Authors:** Yoshinao Ishii, Keiichiro Hayakawa

**Affiliations:** Toyota Central R&D Labs., Inc., 41-1, Yokomichi, Nagakute, Aichi 480-1192 Japan

**Keywords:** Environmental sciences, Environmental social sciences, Geography, Geography, Social sciences

## Abstract

Understanding the relationship between the built environment and urban vibrancy is crucial for effective urban planning and policy development. While recent research using big data and regression analysis has identified built environment factors associated with vibrancy, most studies focus on densely populated urban cores. However, many cities now contain both thriving centers and depopulated areas, which pose sustainability challenges and require targeted strategies. This study investigates whether conventional big data driven approaches can provide reliable, context-sensitive insights when applied to a city encompassing both urbanized and depopulated areas, particularly under data limitations. Using Toyota City, Japan, as a case study, we employed large-scale GPS trajectory data as a proxy for human activity and constructed built environment factors from readily available Geographic Information System data, including land-use maps, building-use/stock information, road and railway networks, and point of interest (POI) locations, to quantify key dimensions of the built environment, namely diversity, density, and accessibility. Global and local regression models were applied to analyze spatial variation in relationships between vibrancy and built environment factors. The results show that these relationships differ markedly between urbanized and depopulated areas; for example, POI density correlates strongly with vibrancy in urbanized areas, whereas residential density is more critical in depopulated areas. These findings demonstrate that big data driven vibrancy analysis can yield meaningful insights even in data-scarce contexts, extending its applicability to diverse urbanized-depopulated settings.

## Introduction

Urban vibrancy, a concept first introduced by Jacobs in 1961^1^, has been a subject of extensive research owing to its profound impact on urban environments^2–4^. Urban vibrancy generally refers to the attractiveness, diversity, and vitality of urban spaces generated by human activities and interactions^4,5^. Previous research has demonstrated that promoting urban vibrancy significantly contributes to revitalizing local economies, fostering community building, improving public safety, and enhancing sustainability^6^. Consequently, analyzing cities through the perspective of vibrancy is crucial for understanding their current state and formulating effective urban development strategies.

A growing body of research has established a strong link between urban vibrancy and the built environment^7^. Specifically, factors such as place diversity, density, and accessibility have been identified as key determinants of vibrancy^8^. Early investigations into urban vibrancy primarily relied on qualitative and observational approaches, which later evolved to include quantitative methods such as surveys and fieldwork^1,9,10^. While these methods offered valuable insights through direct observation, their limitations in terms of spatial and temporal coverage owing to data collection constraints have prompted a shift towards more quantitative approaches.

Recent advancements in information and communication technologies (ICTs) and sensing technologies have enabled the acquisition of large-scale human-activity data, such as GPS trajectories, smart card records, and social media data, with high spatial and temporal resolution^11,12^. Coupled with the increasing availability of comprehensive geospatial datasets, including land-use maps, residential density information, and detailed street networks^13^, these advancements have facilitated a new wave of research. This involves utilizing big data to analyze urban vibrancy at the city scale, employing human activity data as proxies for vibrancy and deriving influencing factors related to the built environment from geospatial data^6,8,14–17^.

However, despite the growing body of literature on urban vibrancy, a significant gap remains. Most big data driven studies have incorporated spatial heterogeneity in analyzing urban vibrancy, typically by comparing conditions between urban cores and their surrounding areas. While these approaches have provided valuable insights, they have largely focused on highly urbanized settings, where the diversity of land use and the coexistence of different demographic dynamics are limited. Consequently, big data driven studies have predominantly concentrated on densely populated urban centers where data collection is relatively straightforward. Meanwhile, many cities worldwide are experiencing population declines in some areas and increasing concentrations in their cores, leading to the expansion of depopulated areas^18–20^. These depopulated areas present unique challenges for cities encompassing both dense urban districts and sparsely populated areas within their administrative boundaries. Depopulated areas pose significant sustainability challenges, necessitating targeted strategies to enhance their vibrancy^21,22^. Nonetheless, studies examining urban vibrancy in cities with both urbanized and depopulated areas using big data approaches remain limited.

This study aims to clarify the applicability of conventional big data driven urban vibrancy analysis in mixed urbanized-depopulated contexts. To this end, we use Toyota City, Japan, which contains both dense urbanized districts and depopulated areas within a single municipality, as a case study to investigate overall trends in urban vibrancy and the distinct characteristics of these contrasting areas. We use comprehensive GPS trajectory data to estimate population presence as a proxy for urban vibrancy (dependent variable). Built environment factors (independent variables) are constructed from readily available Geographic Information System (GIS) data, including land-use maps, building-use/stock information, road and railway network data, and point of interest (POI) locations, to operationalize key dimensions of the built environment–diversity, density, and accessibility. Specifically, we use land-use mix, residential density, POI density, road and railway density, and natural and undeveloped land density. We then conduct regression analyses using both ordinary least squares (OLS) and geographically weighted regression (GWR) models. This approach allows for both global and local analyses of the influence of built environment factors on vibrancy at both citywide and local scales.

Our findings reveal significant variations in the impact of built environment factors on vibrancy. Specifically, land-use mix does not contribute to vibrancy, whereas the mixed use of amenity-related POIs is strongly correlated with increased vibrancy. The local analysis further indicates substantial differences in coefficients between urbanized and depopulated areas, highlighting the need for spatially nuanced planning strategies. In addition, our budget-neutral scenario analysis illustrates how urbanized and depopulated areas can be jointly considered to derive resource allocation strategies, providing practical guidance for balancing efficiency and sustainability. By broadening the scope of urban vibrancy, this research contributes to a deeper understanding of urban vibrancy dynamics in diverse urban contexts and provides valuable insights for policymakers and urban planners.

The remainder of this paper is organized as follows. Following this introduction, the literature on urban vibrancy and the challenges of depopulated areas is reviewed. Next, the data sources and the regression methodologies employed in the study are described. This is followed by the results and discussion of the global and local analyses, alongside a budget-neutral scenario analysis. Finally, the paper concludes with a summary of the key findings and a discussion of the limitations and policy implications of this research.

## Literature review

### Urban vibrancy

The concept of urban vibrancy, first introduced by Jacobs in 1961^1^, describes the richness of human activity and the vitality generated by the diverse interactions and uses within urban spaces. Jacobs’ foundational work emphasized the importance of diversity and spontaneous interactions within urban for fostering safe and thriving urban environments, laying the groundwork for subsequent research on urban vibrancy.

Early investigations into urban vibrancy primarily relied on qualitative observations, surveys, and interviews, including field observations and social surveys, using indicators such as pedestrian counts, durations of public space usage, and diversity of users^9,10^. These methods, while enabling direct observation of vibrancy indicators such as pedestrian counts, public space usage, and user diversity, were often limited by subjectivity, cost constraints, and the challenges of collecting large-scale data. This has boosted the demand for more precise and extensive quantitative methods^7^.

The emergence of GIS marked a significant shift towards quantitative analysis in urban vibrancy research. GIS enabled researchers to quantify spatial factors influencing vibrancy, such as land-use diversity, building density, and accessibility^23,24^. By analyzing the spatial distribution of vibrancy-related factors, researchers could investigate the correlations between physical attributes such as street connectivity and building density with urban vibrancy^25,26^. These studies provided empirical support for Jacobs’ theories, demonstrating that areas with mixed land use and highly connected street networks tend to exhibit higher levels of vibrancy.

### Big data driven urban vibrancy analysis

Recent advancements in information and communication technologies (ICT) and sensing technologies have enabled the use of big data that directly captures human activities in urbanized areas with high spatiotemporal resolution and extensive coverage. By utilizing activity intensity indicators derived from such big data as proxies for urban vibrancy, an increasing number of studies have conducted city-scale vibrancy analyses in recent years^15,17^.

A typical big data driven urban vibrancy analysis generally follows the process outlined below. First, vibrancy indicators are calculated for each spatial unit based on big data sources. Next, potential influencing factors of vibrancy within the target city are appropriately selected and computed using data such as GIS datasets. Finally, regression models are applied using the vibrancy indicators as dependent variables and the selected factors as independent variables, in order to statistically evaluate the effects of each factor on urban vibrancy. In what follows, we review existing studies corresponding to each step of this process and clarify the positioning of the present study.

Commonly used big data sources in vibrancy research include GPS trajectory data^27,28^, smart card data^29^, and social media data^30^. Activity indicators such as the number of people staying in or visiting specific spatial areas, derived from these datasets, are frequently adopted as direct proxies for urban vibrancy. Some studies rely solely on indicators derived from big data, while others combine them with conventional indicators such as urban facilities or economic statistics. These big data sources can be subject to sampling bias, potentially underrepresenting specific groups (e.g., older adults and tourists) and causing discrepancies from actual urban activity patterns^31,32^. Nevertheless, their unprecedented spatiotemporal coverage enables citywide vibrancy analysis, and findings based on such proxies are often broadly consistent with Jacobs’ theories. Because each source captures different aspects of activity, data selection should follow the research objective. GPS trajectory data provide time-ordered information on mobility and stays but not the type or purpose of activities, whereas smart card and social media data more readily reflect activity or consumption types but typically offer limited information on movements before and after the recorded events.

A wide variety of factors have been employed in big data driven vibrancy analyses, typically selected from those identified in prior urban vibrancy studies and adapted to the context of the target city. In most studies, built environment factors available from recently developed GIS datasets are commonly used at the city scale. Following the tradition of classical urban vibrancy research, built environment factors are generally categorized into three dimensions: diversity, density, and accessibility^8^. Representative factors include: land-use mix and POI mix for diversity; floor area or density of residential and office buildings or POIs for density; and distance to the nearest station, total road length, and number of intersections for accessibility.

To ensure interpretability, regression models are often employed to analyze the influence of each factor on urban vibrancy. The OLS model is widely used to capture citywide trends^17^, while the GWR model is applied to reveal spatial heterogeneity^6^. For spatiotemporal variations, the geographical and temporal weighted regression (GTWR) model is used in some studies^27^.

In previous studies, the focus was primarily on urbanized areas where abundant data is available, allowing the inclusion of a wide range of variables. However, when depopulated regions are included in the analysis, data availability is often limited, restricting the number of variables that can be considered. This study aims to demonstrate that, even when analyzing a city that includes depopulated areas using only a limited number of variables derived from publicly available data, the conventional urban vibrancy analysis framework remains functional and can still yield meaningful insights to support appropriate urban development decisions.

### Increase in depopulated areas

The global trend of urbanization, characterized by increasing population concentration in urban centers, is often accompanied by a decline in rural populations^19^. This phenomenon is particularly pronounced in regions such as Europe, the United States, and East Asia, where rural populations are projected to decrease significantly^19^. In Japan, this trend is further exacerbated by a nationwide population decline, with mountainous rural villages experiencing the most severe depopulation impacts^33^. Addressing the challenges posed by these depopulated areas is crucial for ensuring the long-term sustainability of cities and regions. These challenges include declining economic activity, aging populations, and the erosion of social infrastructure. Enhancing vibrancy in these areas is essential to mitigate these negative impacts and promote revitalization^21,34^.

Numerous studies have explored strategies to address the unique challenges of depopulated regions^22,35^. These encompass diverse approaches, including participatory planning involving local residents, leveraging tourism and green infrastructure development, and restructuring policies to stimulate economic revitalization.

While these studies provide valuable insights for tackling depopulation, there remains an important gap in understanding how big data can be utilized to analyze and enhance vibrancy in cities with depopulated areas. Most existing big data driven urban vibrancy studies have considered spatial heterogeneity by comparing urban cores and their surrounding areas, but few have jointly examined both urbanized and depopulated regions within the same analytical framework. This study aims to bridge this gap by analyzing the factors influencing vibrancy in a Japanese city characterized by both urbanized and depopulated areas. By employing big data and traditional regression analysis, we seek to expand the applicability of urban vibrancy research to diverse urban contexts.

## Data and methods

### Study area

**Fig. 1:**
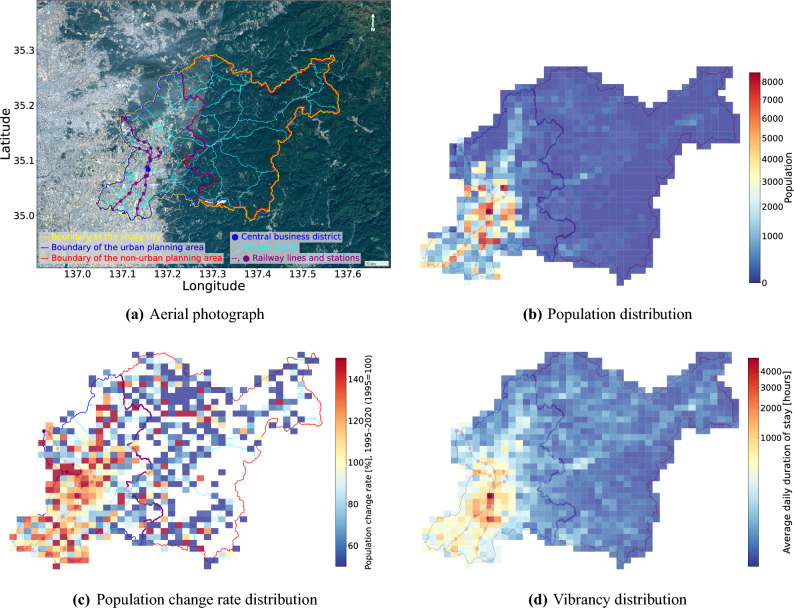
Indicators of the study city: Toyota City, Japan. The aerial photograph is created by editing the Aerial Photograph of Geospatial Information Authority of Japan https://mapps.gsi.go.jp/maplibSearch.do. Source of the administrative and planning boundary shape information and the railway network information (throughout this paper): National Land Numerical Information Download Site of Ministry of Land, Infrastructure, Transport and Tourism (MLIT) of Japan https://nlftp.mlit.go.jp/ksj/. The arterial road network information (throughout this paper) is derived from OpenStreetMap (©OpenStreetMap contributors)^36^.

This study focuses on Toyota City in Aichi Prefecture, Japan, as the target for analysis. Toyota City, the largest city in Aichi Prefecture, spans 918.32 km^2^ and has a population of 422,330 (as of 2020), making it the second most populous city in the prefecture. Figure 1a shows an aerial photograph of Toyota City along with its 2024 administrative boundary and the arterial road and railway networks. Notably, Toyota City is divided into a western urban planning area (delineated by the blue line) and an eastern non-urban planning area (delineated by the red line), a division that facilitates more intensive urbanization within the former. Note that these features are overlaid in the background of subsequent figures to enhance the interpretability of the spatial patterns. Figure 1b illustrates the 2020 population distribution within 1 kilometer square grid cells (hereafter referred to as 1 km cells) that include the city^37^. As depicted in the figures, Toyota City exhibits a distinct spatial pattern: a densely populated urban area concentrated in the western urban planning area and a sparsely populated, expansive mountainous and forested region in the eastern non-urban planning area. Figure 1c presents the population change for each cell between 1995 and 2020, expressed as the percentage of the 1995 population (1995=100%), using 1995 census data^37^ as the baseline. While the city’s total population increased by 10% over this 25-year period, many cells within the mountainous and forested areas experienced population decline. This coexistence of population growth and decline within a single city makes Toyota City a suitable case study for investigating urban vibrancy in the context of increasing depopulated areas globally.

This study employs a vibrancy analysis using big data driven regression methods. All variables for the analysis are defined at the 1 km cell level to align with the spatial resolution of the independent variable data source.

### Dependent variables

Following established practices in big data driven urban vibrancy studies, this study adopted mobile phone record counts derived from GPS trajectory data as the measure of vibrancy (dependent variable) for each cell^6^. Given the likely lower prevalence of smart card use and check-in activity in depopulated areas, we adopted GPS trajectory data, which is expected to provide comparatively higher coverage in such contexts. The GPS trajectory data used in this analysis were provided by Agoop Corp., a subsidiary of Japan’s largest telecommunications carrier. Agoop data, characterized by high population coverage and a sampling time of approximately 1 min, is widely used for mobility analysis in Japan^38,39^. For privacy protection, Agoop data includes only records from consented users and contains no direct personal identifiers (e.g., names or home addresses). Device identifiers are hashed and rotated daily, and points within 100 m of each user’s estimated home location were removed prior to data provision, substantially reducing re-identification risk.

This study uses Agoop data collected between July 1, 2023, and June 30, 2024, a one-year period after the effects of reduced mobility due to COVID-19 had subsided. The dataset includes latitude, longitude, and timestamp information for all users passing through the analysis area during this period. Using this data, the total mobile phone record counts for each 1 km cell–representing the cumulative time users spent in each cell were calculated and used as the vibrancy indicator (dependent variable) for that cell.To focus on mobility-related activity and avoid unreliable stay estimations, we excluded periods before a user’s first movement and after their last movement of the day. Since data within 100 m of estimated home locations were removed prior to provision for privacy protection, these excluded periods likely correspond to unobserved stays at home. By excluding them, we ensure that the vibrancy indicator reflects only manifest activity recorded between the first and last movements, rather than inferred stays in unobserved home areas. Table 1 presents an overview of the dependent variable, and Fig. 1d shows its spatial distribution.

The calculation of global Moran’s I, with adjacent cells defined as the eight surrounding cells, yielded a value of 0.619 (p-value = 0.001), indicating a statistically significant spatial dependency in vibrancy distribution within the city. Interestingly, the distribution of stay time exhibits a greater concentration in the central business district (CBD) compared to the population distribution. This can be attributed to the city’s daytime population being 1.1 times larger than its nighttime population and the presence of numerous facilities and POIs that attract people to the CBD.

**Table 1:** Summary of dependent variable.

Variable	Definition	Data source	min	max	mean	SD
Vibrancy	Average daily time (hours) spent by mobile phone users	GPS trajectory data from Agoop Corp.	0.001	4,962.2	112.4	326.4

### Independent variables

**Table 2:** Summary of independent variables.

Variables	Types	Definition	Data source	min	max	mean	SD
Land-use mix	Diversity	Entropy of distribution of residential, commercial, and office	Building Statistics Data from Zenrin Co., Ltd.	0.0	1.0	0.04	0.09
POI density	Density	Number of POI units	Building Statistics Data from Zenrin Co., Ltd.	0	974	7.19	36.52
Residential density	Density	Number of residential units	Building Statistics Data from Zenrin Co., Ltd.	0	4,857	200.3	530.0
Road and railway density	Accessibility	Proportion of road and railway area to the cell area	Land Use Classification Data from database of MLIT of Japan	0.0	0.28	0.01	0.03
Natural land density	Density, accessibility	Proportion of natural land area to the cell area	Land Use Classification Data from database of MLIT of Japan	0.0	1.0	0.74	0.31
Convertible land density	Density, accessibility	Proportion of convertible land area to the cell area	Land Use Classification Data from database of MLIT of Japan	0.0	0.89	0.11	0.14

This section describes the built environment factors (independent variables) used in this study. Table 2 summarizes these variables. Each variable is explained in detail in the following subsections.

#### Land-use mix

Land-use mix, a widely used indicator of diversity within an area, was employed in this study. Studies on land-use mix in Japan have often used the “Urban Planning Decision Information Data” GIS dataset provided by local governments^40^, which categorizes areas by use–such as residential, commercial, and industrial–and serves as a common basis for calculating the degree of land-use mix^40,41^. However, this dataset is limited to urbanized areas and excludes many depopulated areas in the target city that fall outside the scope of urban planning zones. Consequently, it is not feasible to obtain the area of each land-use category for the entire city.

To overcome this limitation, this study approximates land-use mix using the “Building Statistics Data” for 2020 provided by Zenrin Co., Ltd. This dataset, commonly used in geospatial studies in Japan^41,42^, provides information on the number of residential units, commercial facilities, and offices within each 1 km cell. The degree of mixture among these categories serves as a proxy for land-use mix. Following previous research, Shannon’s entropy was used to quantify the level of mixture^6^. This approach allows for a consistent and scalable approximation of land-use diversity across both urbanized and depopulated areas of a target city.

#### POI density

As research on urban vibrancy using big data has progressed, it has become increasingly clear that POI density also has a significant influence on vibrancy^8,15^. In this study, POI density is adopted as an indicator of density within an area. For each 1 km cell, the numbers of POIs belonging to the following ten categories—“restaurant,” “shop,” “service,” “bank,” “infrastructure,” “sport,” “entertainment,” “hotel,” “healthcare,” and “social”—are counted, and their total is used to define the POI density. As with the land-use mix, the data source for POI counts is the 2020 Building Statistics Data.

#### Residential density

Following the approach of Frank *et al*^43^, this study uses residential density as an indicator of density within each cell. Residential density is measured as the number of residential units per 1 km cell, based on the 2020 Building Statistics Data. Because the cell areas are nearly identical, the number of residential units per cell serves as a direct measure of residential density.

#### Road and railway density

To assess accessibility within each cell, this study adopts the proportion of road and railway area to the total cell area, following the approach of Tu *et al*^6^. According to the most recent Person Trip Survey available for Toyota City (2011), approximately 80% of trips were made by rail and private car, with private car trips accounting for more than 70%^44^. Given this mobility structure, we adopt road and railway area proportions as parsimonious proxy variables for mobility-related accessibility in this study. These area proportions are derived from the 2021 “Land Use Classification Data” published by the Ministry of Land, Infrastructure, Transport, and Tourism (MLIT), Japan^45^. This dataset classifies land use types for 100-m cells across Japan based on satellite imagery including categories such as building sites, roads, railways, and various types of natural or convertible land. The proportion of road and railway area within each 1 km cell serves as a proxy for accessibility, reflecting the ease of movement within the cell.

#### Natural land density and convertible land density

This study incorporates two variables to capture the characteristics of non-built-up land: *natural land density* and *convertible land density*. Natural land density represents the proportion of areas, namely forests, rivers and lakes, beaches, and seawater. These areas are not generally developable and primarily serve ecological and recreational functions. Convertible land density represents the proportion of land types, namely paddy fields, other farmland, and wasteland. Unlike natural land, these areas can be more feasibly converted into other uses through urban planning, such as residential or POI development, and thus have direct implications for local vibrancy when considering redevelopment or land-use change.

As shown in Fig. 1a, both natural land and convertible land are particularly prevalent in depopulated areas, where they are expected to correlate with building density and transportation accessibility. Distinguishing between these two categories allows us to better capture their different roles in shaping vibrancy. Each density is calculated as the proportion of the respective land category within each 1 km cell, derived from the Land Use Classification Data introduced in the previous section.

### Regression analysis

This study employed regression analysis to examine the influence of the defined independent variables on urban vibrancy. Two approaches were used: a global regression analysis to capture citywide average effects and local regression analysis to reveal spatial heterogeneity within the relationships. Prior to the analyses, all variables were standardized to have a mean of 0 and a variance of 1.

#### Global regression analysis

An OLS model was used for the global regression analysis to estimate the coefficients of each factor across the city as follows:

1 $$\begin{aligned} y = \beta _0 + \sum _{k=1}^{K} \beta _k x_{k} + \epsilon , \end{aligned}$$

where *y* is the dependent variable, $x_k$ is the *k*-th independent variable, $\beta _0$ is the intercept term, $\beta _k$ is the regression coefficient for the *k*-th variable, *K* is the total number of independent variables, and $\epsilon$ is the error term.

#### Local regression analysis

To capture the spatial heterogeneity in the relationships between urban vibrancy and the independent variables, GWR was employed. Temporal heterogeneity analysis was challenging due to the sparsity of vibrancy indicators in depopulated areas, and it was necessary to aggregate data over time to reduce this sparsity. GWR is a widely used method for analyzing spatial variations in relationships and is commonly applied in urban vibrancy studies^46^.

The GWR model estimates the relationship between the dependent variable $y_i$ and explanatory variable $x_i$ at a specific spatial location *i* is expressed as

2 $$\begin{aligned} y_i = \beta _0(u_i, v_i) + \sum _{k=1}^{K} \beta _k(u_i, v_i) x_{ik} + \epsilon _i, \end{aligned}$$

where $u_i$ and $v_i$ are the spatial coordinates (latitude and longitude) of location *i*, allowing different regression coefficients $\beta _k(u_i, v_i)$ to be estimated for each location. The GWR model uses weighted least squares with a Gaussian kernel function to estimate the coefficients. The optimal bandwidth parameter for the kernel is determined based on the corrected Akaike Information Criterion. The spatial coordinates $u_i$ and $v_i$ are represented by the latitudes and longitudes of the centroids of each cell, respectively. The model was implemented using the Python library *mgwr* (version 2.2.1)^47^.

## Results and discussion

### Results and discussion of global regression analysis

Table 3 presents the results of the global regression analysis obtained using the OLS model (1). Note that “SD” in the table denotes the standard deviation. The adjusted R$^2$ of 0.816 indicates that the selected independent variables effectively explain the variation in urban vibrancy, supporting the robustness of the model. Furthermore, the variance inflation factors (VIFs) for all independent variables were calculated to assess multicollinearity. The maximum VIF value of approximately 2.28 indicates that multicollinearity is not a concern in this analysis.

**Table 3:** Results of global regression analysis.

Variables	Coefficient	*T*-value	SD
Constant	−0.000	-	0.014
Land-use mix	0.012	0.831	0.015
POI density	0.526	28.625***	0.018
Residential density	−0.053	−1.980*	0.027
Road and railway density	0.074	4.101***	0.018
Natural land density	−0.726	−19.564***	0.037
Convertible land density	−0.375	−14.856***	0.025

*** significant at the 0.001 level, * significant at the 0.05 level.

Interestingly, land-use mix does not exhibit a significant association with vibrancy in the target city. This unexpected finding may be attributed to the unique characteristics of the study area, which includes a large proportion of natural or convertible land. In such areas with limited buildable land, increasing the diversity of the existing buildings may not be sufficient to attract people and enhance vibrancy. This observation aligns with recent research questioning the universal positive impact of land-use mix on vibrancy^8,48,49^. This highlights the importance of considering the context of cities with depopulated areas.

By contrast, POI density, which reflects the density and availability of amenities, shows a significantly strong positive association with vibrancy across the city. This finding suggests that areas with a greater number of amenity-related facilities, which serve as potential destinations, are more likely to attract people and generate higher levels of vibrancy. Therefore, in citywide analyses that include depopulated areas, increasing the number of amenity POIs in each area, rather than focusing solely on improving land-use mix, may be a more effective strategy for enhancing urban vibrancy.

However, residential density shows a negative association with vibrancy. This unexpected result suggests that, at the city scale, increasing population density through residential development may not be as effective in enhancing vibrancy as increasing the availability of destination facilities like amenities. This finding could be attributed to the functional characteristic of the target city, which serves primarily as a workplace rather than a residential area, as evidenced by its larger daytime population. Road and railway density, representing accessibility, has a significant positive association with vibrancy. This highlights the crucial role of transportation infrastructure in facilitating movement and attracting visitors, ultimately contributing to increased vibrancy in both urbanized and depopulated areas.

Finally, both natural land density and convertible land density show strong negative associations with vibrancy. Natural land density exhibits a particularly large negative effect, reflecting the limited accessibility and development potential of such areas. Convertible land density also shows a significant negative effect, indicating that the presence of unused land suppresses vibrancy at the citywide level. Such convertible land may contribute to improved vibrancy if redeveloped for other uses through urban planning, but any land-use change needs to be carefully assessed in light of planning objectives and development costs. With respect to natural land, it is not appropriate to treat all such areas uniformly as targets for vibrancy-oriented development; rather, the decision of whether and where to develop should be made selectively and cautiously, considering diverse perspectives such as conservation, disaster prevention, and recreational value.

### Results and discussion of local regression analysis

**Table 4:** Results of local regression analysis.

Variables	Mean	SD	Min	Max	Mean abs. local *T* value
Constant	−0.161	0.104	−0.572	−0.027	2.50
Land-use mix	0.022	0.034	−0.003	0.145	0.87
POI density	0.326	0.167	0.088	0.569	11.50
Residential density	0.093	0.198	−0.400	0.333	2.75
Road and railway density	0.093	0.079	−0.046	0.271	1.91
Natural land density	−0.371	0.500	−1.615	−0.010	6.62
Convertible land density	−0.190	0.257	−0.886	−0.003	5.32

**Fig. 2:**
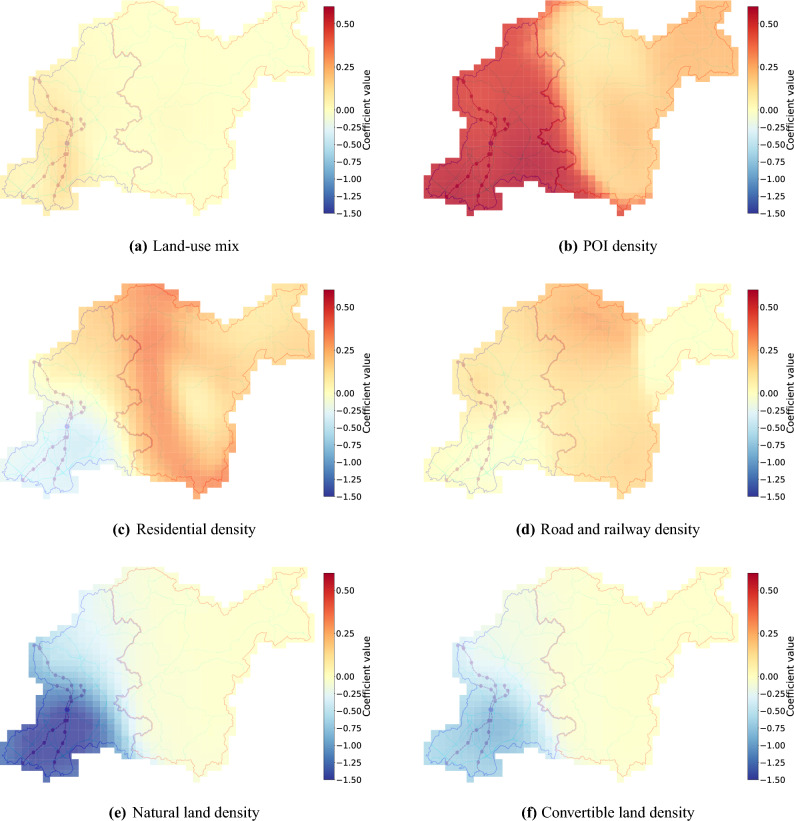
Spatial distribution of coefficients by local regression analysis.

Table 4 lists the coefficients obtained from the GWR model (2). The adjusted R$^2$ of 0.878, which is very high, reinforces the robustness of the model and its ability to capture spatial variations in the relationships between urban vibrancy and the independent variables. Additionally, Fig. 2 visually depicts the spatial distribution of these coefficients, revealing significant heterogeneity in their influence on vibrancy (see Fig. 1 and the overlays in Fig. 2 for the distribution of urbanized and depopulated areas). As noted in an earlier section, the global Moran’s I of 0.619 for the dependent variable indicates high spatial dependence, which is reflected in the substantial spatial variations observed for several variables. The spatial distributions of the coefficients for each independent variable are discussed below.

The spatial distribution of coefficients for land-use mix (Fig. 2a) reveals an interesting pattern. Consistent with the global regression analysis, the coefficients remain close to zero across most of the city, indicating that land-use mix has a limited influence on vibrancy overall. However, a notable exception is observed near the CBD, where the coefficients show a significantly positive value of up to approximately 0.15. This suggests that while land-use mix may not be a significant driver of vibrancy in areas with limited buildable land, such as depopulated areas, it can contribute to vibrancy in already thriving urban centers. This finding highlights the importance of considering the existing context and vibrancy levels of specific areas when applying land-use mix strategies in cities with both urbanized and depopulated areas.

The spatial distribution of coefficients for POI density (Fig. 2b) further illustrates the strong and spatially varying influence of POI concentration on urban vibrancy. As noted earlier, the global analysis showed a significant positive association between POI density and vibrancy across the city. The local analysis confirms this trend, indicating a generally positive relationship throughout the city, with particularly strong positive effects observed in urbanized areas. This result suggests that in urbanized areas, where accessibility from surrounding cities is relatively high, a high concentration of POIs plays an important role in attracting people and generating vibrant activity. In contrast, in depopulated areas, the effect of POI density on vibrancy is considerably weaker. This may be due to poor transportation access and low population density, which limit the extent to which an increase in POIs can enhance vibrancy. These findings highlight the importance of considering spatial differences in the effects of density-related factors when developing strategies to improve urban vibrancy.

The spatial distribution of coefficients for residential density (Fig. 2c) presents a compelling contrast between urbanized and depopulated areas. In urbanized areas, residential density exhibits a negative association with vibrancy, suggesting that increasing the number of residents may not be the most effective strategy for enhancing vibrancy in these already densely populated areas. Instead, the focus should be on diversifying destination facilities and amenities, such as POIs, to attract visitors and support a wider range of activities. Conversely, in depopulated areas, residential density shows a positive association with vibrancy. This implies that increasing residential density to boost population may be crucial for revitalizing these areas, as a critical mass of residents is needed to support local businesses, services, and community activities. This contrasting pattern highlights the need for tailored approaches to urban planning that consider the unique needs and characteristics of different areas within a city.

The spatial distribution of coefficients for road and railway density (Fig. 2d) reinforces the importance of accessibility in shaping urban vibrancy. Accessibility generally exhibits a positive association with vibrancy across the city, except in the sparsely populated northeastern areas. Interestingly, the northern areas, characterized by dispersed settlements, show particularly large coefficients. This suggests that road infrastructure plays a crucial role in connecting these dispersed communities and facilitating access to activities and services, thereby contributing significantly to vibrancy. These findings demonstrate the value of spatial analysis using the GWR model in identifying areas where improvements in accessibility are most needed to enhance vibrancy, particularly in depopulated areas with geographically dispersed settlements.

The spatial distributions of coefficients for natural land density and convertible land density (Figures 2e and 2f) indicate negative impacts on vibrancy in urbanized areas, while their effects are negligible in depopulated areas. The negative effect is stronger for natural land, suggesting that its presence in dense urban contexts is particularly associated with reduced vibrancy. These results highlight the importance of reducing the share of non-built-up land and optimizing land use in urbanized areas. In particular, for the city examined here, decreasing natural land was associated with substantial gains in vibrancy; however, such changes must be carefully evaluated in light of potential impacts on the environment and landscape. Therefore, land-use strategies should take advantage of redevelopment opportunities for convertible land while maintaining a balance with the conservation of natural land.

To further validate these local insights against concrete built environment factors, we examined the specific cell where the CBD is located in the western urban planning area (refer to Fig. 1a and the overlays in Fig. 2). This cell represents the city’s most intensive built environment, where land-use mix, POI density, residential density, and road and railway density all fall within the top 1% citywide, while natural and convertible land densities are within the bottom 1%. In this high-density context, the local coefficients for land-use mix (Fig. 2a) and POI density (Fig. 2b) are relatively high compared to the citywide average. This confirms that the concentration and diversity of urban functions are primary drivers of vibrancy in the city’s core. Additionally, the negative local coefficients for natural land density and convertible land density (Figures 2e and 2f) observed in this cell are consistent with the findings for urbanized areas, where the presence of non-built-up land is associated with suppressed vibrancy. Conversely, the negative local coefficient for residential density in this cell (Fig. 2c) aligns with its functional role as a primary workplace hub, where vibrancy is predominantly generated by daytime visitors and workers rather than the resident population. Such alignment between the local coefficients and the actual built environment factors of this highly urbanized cell demonstrates the model’s capacity to yield contextually relevant insights.

#### Budget-neutral scenario analysis using GWR results

Building on the GWR results, this subsection demonstrates how jointly considering urbanized and depopulated areas allows the efficiency of different measures to be compared on a common basis, providing useful insights for resource allocation in urban planning. To this end, we conducted a budget-neutral scenario analysis.

Three policy variables were examined: POI density, residential density, and convertible land density. One intervention unit was defined as adding one POI, adding ten dwellings, or reducing convertible land density by 0.001 (0.1% of a 1 km cell). These units are hypothetical, introduced to compare marginal effects across variables, and should be adjusted to local conditions in practice. Natural areas such as forests and water bodies were excluded, as their treatment is subject to debate. Equal costs were assumed for all intervention types.

Using local coefficients, we calculated the average daily increase in duration of stay (hours) from one unit in each cell. A total budget of 100 units was allocated under four scenarios: investment only in urban POI density, only in residential density in depopulated areas, only in urban convertible land density, or a 50:50 mix of POI and residential. In each scenario, interventions were directed to the top-quartile “effective” cells, beginning with those with the highest expected gain.

Results are shown in Table 5, where #POI, #RES, and #CONV denote the number of intervention units allocated to each variable. The POI density (urbanized only) scenario produced the largest effect, with an average daily increase in duration of stay of 497.3 hours (relative value 100.0). Residential density (depopulated only) yielded 190.6 hours (38.3%), convertible land density (urbanized only) 176.6 hours (35.5%), and the mixed POI + Residential scenario 349.1 hours (70.2%).

These results indicate that strategies for enhancing vibrancy vary substantially with context. POI intensification is most efficient in urban cores, consistent with the strong positive association identified earlier. Residential densification in depopulated areas has limited effects due to a small population base but remains important for community sustainability. Reducing convertible land density yields relatively large absolute gains but involves higher financial and environmental costs, warranting selective implementation. Thus, big data based joint analysis of urbanized and depopulated areas can highlight appropriate resource allocation strategies, offering practical guidance for planners. This integrated approach provides a strong tool for designing urban development that balances efficiency and equity.

**Table 5:** Results of budget-neutral scenario analysis using GWR results.

Scenario	#POI	#RES	#CONV	Average daily increase (hours)	Relative (best=100)
POI density (urbanized only)	100	0	0	497.3	100.0
Residential density (depopulated only)	0	100	0	190.6	38.3
Convertible land density (urbanized only)	0	0	100	176.6	35.5
POI + Residential (urbanized + depopulated)	50	50	0	349.1	70.2

## Concluding remarks

This study examined how built environment factors influence urban vibrancy in a city that encompasses both urbanized and depopulated areas, a context that has received limited attention in previous big data driven studies. By integrating large-scale GPS trajectory data with built environment factors derived from readily available geospatial datasets, we explored both citywide and spatially varying relationships using OLS and GWR models. The results revealed substantial heterogeneity in the determinants of vibrancy. While POI density and transportation accessibility consistently enhance vibrancy across the city, residential density plays contrasting roles between urbanized and depopulated areas. In urbanized areas, higher POI density strongly correlates with greater vibrancy, suggesting that the concentration of amenity-related facilities attracts more human activity. In contrast, in depopulated areas, increasing residential density appears more important for sustaining local vibrancy, as a minimum population presence is essential to support community functions and local services. Land-use mix showed little influence in either context, indicating that the conventional assumption of its universal positive effect may not hold in cities with both urbanized and depopulated areas. These findings highlight the importance of context-sensitive urban planning strategies that account for spatial heterogeneity in cities experiencing demographic polarization.

Despite these insights, several limitations should be acknowledged. First, land-use mix was calculated based on the distribution of building types rather than conventional land-use areas, due to the absence of comprehensive land-use data in depopulated areas. While this proxy enabled citywide analysis, it may differ from traditional measures that rely on officially zoned land categories. Future studies could improve the precision of diversity indicators by incorporating open-source geospatial datasets such as OpenStreetMap^36^, which are becoming increasingly available as data infrastructure expands.

Second, the vibrancy indicator derived from GPS trajectory data may be subject to sampling bias and thus may not fully represent all demographic groups or activity patterns. Because inclusion depends on smartphone usage and location-sharing consent, the data may underrepresent populations such as older adults, individuals with lower smartphone usage, or residents in areas with limited connectivity, and the degree of underrepresentation may differ between urbanized and depopulated areas. In addition, while GPS-derived presence captures where and when people are observed, it does not directly indicate the type or purpose of activities. Therefore, our results should be interpreted as capturing a key dimension of urban vibrancy, namely population presence and exposure, while recognizing that GPS data do not directly reveal the type or purpose of activities. Future work could strengthen validity and assess potential biases by triangulating with complementary data sources such as smart card records for public-transport use, social media activity for social engagement, census statistics for demographic structure, or other sensor-based data, to provide a more comprehensive and demographically balanced picture of urban vibrancy.

Third, accessibility was operationalized using road and railway area proportions as parsimonious proxies for transport infrastructure provision, and therefore does not capture multimodal mobility conditions such as walkability, cycling infrastructure, public transport service levels, or first and last mile connectivity. In addition, POI density and natural land density may be interrelated, particularly in regions where topography constrains development, which may complicate the interpretation of their separate associations with vibrancy. Moreover, because demographic attributes are not available in the GPS dataset, we could not examine how mobility behavior and observed vibrancy may vary across demographic groups. Future work could address these limitations by incorporating demographic statistics and multimodal accessibility indicators and by explicitly modeling potential dependencies between POI density and natural land, for example through interaction terms or topographic controls.

Fourth, while Toyota City serves as an illustrative case of demographic polarization in Japan, the generalizability of certain results should be interpreted within its specific geographical and functional context. For instance, the negative association between residential density and vibrancy in urbanized cells may be context-specific, reflecting Toyota City’s unique role as a major industrial hub with a high daytime-to-nighttime population ratio. Similarly, the pronounced influence of road infrastructure in northern areas is closely linked to the dispersed settlement patterns, mountainous topography, and the high automobile modal share characteristic of car-dependent regional Japan.

Even with these limitations, the study provides meaningful implications for both research and practice. The fundamental insight that vibrancy determinants are spatially heterogeneous–where POI density drives vibrancy in urbanized cores while residential density is critical for sustaining the minimum population presence required to support community functions and local services in depopulated peripheries–provides a highly transferable framework for targeted urban planning. Furthermore, our integrated approach of jointly evaluating urbanized and depopulated areas within a single analytical framework is broadly applicable, offering a quantitative method for within-city resource allocation that balances economic efficiency with social equity. Additionally, our results demonstrate that a big data driven approach using parsimonious built environment factors can yield interpretable and contextually relevant insights even in data-scarce or demographically imbalanced environments. By capturing these spatially varying relationships, the application of geographically weighted regression offers a practical tool for developing nuanced policy interventions that balance efficiency and sustainability in diverse urban contexts worldwide.

Overall, this study expands the applicability of big data driven urban vibrancy research beyond densely populated urban cores. As population decline and spatial polarization continue to reshape cities worldwide, understanding how vibrancy emerges and evolves in mixed urbanized-depopulated areas will be essential for achieving more sustainable and resilient urban development.

## Data Availability

The data that support the findings of this study include GPS trajectory data provided by Agoop Corp. and building and point of interest data obtained from Zenrin Co., Ltd., but restrictions apply to the availability of these data, which were used under license for the current study and are therefore not publicly available. Data are however available from the authors upon reasonable request and with permission from Agoop and Zenrin Marketing Solutions. The other datasets generated during and/or analyzed during the current study are available from the “National Land Numerical Information Download Service” of the Ministry of Land, Infrastructure, Transport and Tourism (MLIT) of Japan, the “Portal Site of Official Statistics of Japan (e-Stat)”, and “OpenStreetMap” at https://nlftp.mlit.go.jp/ksj/, https://www.e-stat.go.jp/en, and https://www.openstreetmap.org. The code for data processing, vibrancy metric calculation, and OLS/GWR modeling is available in the Zenodo repository (DOI: https://doi.org/10.5281/zenodo.18740879). This code is provided under a custom license from Toyota Central R&D Labs., Inc.; for specific terms and conditions, please refer to the LICENSE file within the repository. The GPS trajectory data, as well as the building and POI data, are proprietary and are not included in this repository due to licensing restrictions.

## Code availability

The code for data processing, vibrancy metric calculation, and OLS/GWR modeling is available in the Zenodo repository (DOI: https://doi.org/10.5281/zenodo.18740879). This code is provided under a custom license from Toyota Central R&D Labs., Inc.; for specific terms and conditions, please refer to the LICENSE file within the repository. The GPS trajectory data, as well as the building and POI data, are proprietary and are not included in this repository due to licensing restrictions.
